# Phase-dependent Brain Activation of the Frontal and Parietal Regions During Walking After Stroke - An fNIRS Study

**DOI:** 10.3389/fneur.2022.904722

**Published:** 2022-07-19

**Authors:** Shannon B. Lim, Chieh-ling Yang, Sue Peters, Teresa Liu-Ambrose, Lara A. Boyd, Janice J. Eng

**Affiliations:** ^1^Department of Physical Therapy, University of British Columbia, Vancouver, BC, Canada; ^2^Rehabilitation Research Program, GF Strong Rehabilitation Centre, Vancouver, BC, Canada; ^3^Department of Occupational Therapy and Graduate Institute of Behavioral Sciences, College of Medicine, Chang Gung University, Taoyuan City, Taiwan; ^4^Department of Physical Medicine and Rehabilitation, Chang Gung Memorial Hospital, Chiayi, Taiwan; ^5^School of Physical Therapy, Western University, London, ON, Canada; ^6^The David Mowafaghian Centre for Brain Health, University of British Columbia, Vancouver, BC, Canada; ^7^Centre for Hip Health and Mobility, Vancouver Coastal Health Research Institute, Vancouver, BC, Canada

**Keywords:** functional near-infrared spectroscopy (fNIRS), gait, stroke, pre-frontal cortex (PFC), premotor cortex (PMC), sensorimotor cortex (SMC), posterior parietal cortex (PPC)

## Abstract

**Background:**

Recovery of walking post-stroke is highly variable. Accurately measuring and documenting functional brain activation characteristics during walking can help guide rehabilitation. Previous work in this area has been limited to investigations of frontal brain regions and have not utilized recent technological and analytical advances for more accurate measurements. There were three aims for this study: to characterize the hemodynamic profile during walking post-stroke, to investigate regional changes in brain activation during different phases of walking, and to related brain changes to clinical measures.

**Methods:**

Functional near-infrared spectroscopy (fNIRS) along the pre-frontal, premotor, sensorimotor, and posterior parietal cortices was used on twenty individuals greater than six months post-stroke. Individual fNIRS optodes were digitized and used to estimate channel locations on each participant and short separation channels were used to control for extracerebral hemodynamic changes. Participants walked at their comfortable pace several times along a hallway while brain activation was recorded. Exploratory cluster analysis was conducted to determine if there was a link between brain activation and clinical measures.

**Results:**

Sustained activation was observed in the pre-frontal cortex with the ipsilesional hemisphere showing greater activation compared to the contralesional side. Sensorimotor cortex was active during the early, acceleration stage of walking only. Posterior parietal cortex showed changes in activation during the later, steady-state stage of walking. Faster gait speeds also related to increased activation in contralesional sensorimotor and posterior parietal cortices. Exploratory analysis clustered participants into two distinct groups based on their brain activation profiles and generally showed that individuals with greater activation tended to have better physical outcomes.

**Conclusions:**

These findings can guide future research for obtaining adequate power and determining factors that can be used as effect modifiers to reduce inter-subject variability. Overall, this is the first study to report specific oxygenated and deoxygenated hemoglobin changes in frontal to parietal regions during walking in the stroke population. Our results shed light on the importance of measuring brain activation across the cortex and show the importance of pre-frontal, sensorimotor, and posterior parietal cortices in walking after a stroke.

## Introduction

Recovery of walking is a primary rehabilitation goal post-stroke (1), but this recovery tends to be quite variable (2) and the proportion of individuals who regain full community walking function is limited (3). Stroke is a direct injury of the brain, but the functional characteristics of the brain are rarely assessed during the rehabilitation process. With the variability in walking recovery, measuring and documenting brain characteristics can help guide the prescription of rehabilitation treatments (4).

Functional near-infrared spectroscopy (fNIRS) is an increasingly popular tool for measuring brain activation. Its portability, relatively low sensitivity to motion artifacts, and low cost has made it an appealing tool for measuring the brain during walking (5). fNIRS uses paired optodes of near-infrared light emitters and detectors separated by 3–4 cm. This separation distance allows for recording depth of 1.5–2 cm (i.e., reaching the cortical layer of the cerebral cortex) and a relatively high spatial resolution compared to electroencephalography (EEG). These optodes can be placed along a multitude of areas on the scalp to estimate the changes in concentration of both oxygenated (HbO) and deoxygenated (HbR) hemoglobin in the area. Using the neurovascular coupling theory, these changes in hemoglobin concentrations—increased HbO and decreased HbR—indicate increases in cortical brain activation (6, 7).

In healthy adults, the onset of walking from standing typically shows an immediate decrease in HbO (indicating oxygen consumption) followed by an increase (indicating oxygen replenishment/increase oxygen to meet the neuronal demands) that peaks between 5-10s after walking onset. As walking continues, this initial increase in HbO declines, and at times, reaches baseline or below baseline standing levels even before the walking has ceased (8). The response of HbR is typically opposite and the change is comparatively smaller in amplitude. Investigation of the magnitude of activation across the different walking stages (e.g., acceleration or steady-state walking) is important for assessing the relative cortical demand at the different walking stages.

Previous work suggests different hemodynamic response profiles in the stroke population (9). The hemodynamic profiles in the stroke population, however, is limited and a more detailed description of the profiles during walking post-stroke is needed. In stroke, brain activity to date has been primarily measured over the pre-frontal cortex (PFC) due to its accessibility, with fewer studies measuring premotor cortex (PMC), and sensorimotor cortex (SMC) (10). One fNIRS study has specifically shown a sustained elevation in HbO in PFC and supplementary motor area during the entire walking period (11). Overall, the magnitude of activation, especially when the activation is symmetrical, is commonly related to faster and more symmetrical walking post-stroke (12–14). Individuals with more severe hemiparesis also showed greater PFC and PMC activation compared to those with less severe strokes (13). Poor walking performance (i.e., increased stride-time variability) in healthy adults also show increased SMC activation in addition to elevated PMC activity (15). It is currently unclear whether increased activation in all cortical regions is related to stroke severity or if it is beneficial to walking performance after stroke. Additionally, although previous work in both felines (16) and humans (17, 18) have shown that activation in the posterior parietal cortex (PPC) are important during walking, to date, no fNIRS studies have assessed activation in these areas during walking post-stroke. Thus, a more thorough assessment of activation across the cortex and its relation to clinical measures is needed.

Our systematic review showed that the majority of fNIRS studies in stroke have methodological limitations that impact interpretation of the results (10). Notably, none of the studies utilize methods to control for extracerebral changes in hemoglobin, which can significant confound fNIRS signals (7, 19). Short-separation channels, which are optode pairs separated by <1.5 cm, can be used to measure and then remove extracerebral hemoglobin changes and motion artifacts. Additionally, since inter-subject deviations in optode placement can result in measurements from functionally different cortical regions, accurate localization of the optodes is needed. None of the studies utilize methods to improve accuracy, which may impact the overall interpretation of regional activation (20). Thus, using both short-separation channels and individual localization of optodes in a study could improve methodology to a point that increases confidence in brain activity results.

The current study was designed to examine activation patterns across multiple cortical regions and relate activation with stroke impairment and gait performance. Three aims were addressed within this study: 1) to describe the hemodynamic response profile with walking after stroke, 2) to determine if there are regional brain activation differences between phases of walking and 3) to determine if changes in brain activity relate to impairment or walking performance.

We hypothesize that: 1) initial oxyhemoglobin characteristics will decrease at the onset of walking then elevate and remain elevated throughout the entire walking period, 2) cortical regions along the PFC, PMC, SMC, and PPC will increase activation during walking compared to standing, and the specific intensity of activation will be dependent on the phase of walking and the functional role of each region, and 3) a greater magnitude of activation, especially along the ipsilesional hemisphere, will relate to less impairment and better gait performance.

## Methods

### Participants

#### Recruitment

Participants were recruited through purposive sampling via posters at local rehabilitation centers, private clinics, and online platforms. Previous participants who had agreed to be contacted for future studies were also informed about the current study. Study details were approved by the university clinical research ethics board and all participants provided written and informed consent.

#### Screening

Interested individuals were first screened for eligibility via telephone. Inclusion criteria were as follows: age greater or equal to 18 years old; telephone-Mini-Mental Status Exam (MMSE) score of <21/26 (21) indicating mild cognitive impairment at most; single known stroke incident <6 months previous (chronic stroke); one-sided hemiparesis; able to walk independently (gait aids allowed) for continuous 1-min bouts; and able to understand and follow directions in English. Exclusion criteria were orthopedic injury impairing current walking and neurological injury other than stroke.

#### Sample Size Calculation

With data from previous work using fNIRS to examine pre-frontal cortex brain activation during walking in the stroke population (22), a sample size of 21 participants was estimated to identify a difference between standing and normal-paced walking (power 80%, alpha = 0.05). No data were available to calculate sample size estimates based on other brain regions during walking in the stroke population.

#### Demographic Data

Age, sex, gait aid used, and global cognition (using the Montreal Cognitive Assessment Tool) were collected. Stroke characteristics were obtained through medical charts when available. When charts were not available, details on time post-stroke, and stroke type (ischemic/hemorrhagic) were collected through verbal reports by the participants. Lesion location was determined through structural MRI obtained through medical records or collected for this study when eligible.

### Measures

#### Functional Brain Activation

Functional brain activity was measured using fNIRS. Each participant's head circumference was measured to fit the headcap (EASYCAP GmbH, Germany) that was populated with fNIRS optodes. The Cz position [10/20 International, (23)] was measured by marking the intersection between nasion-inion and periauricular points. The cap was then placed on the participant's head with reference to Cz and visually inspected for alignment along the midsagittal plane.

**Figure 1:**
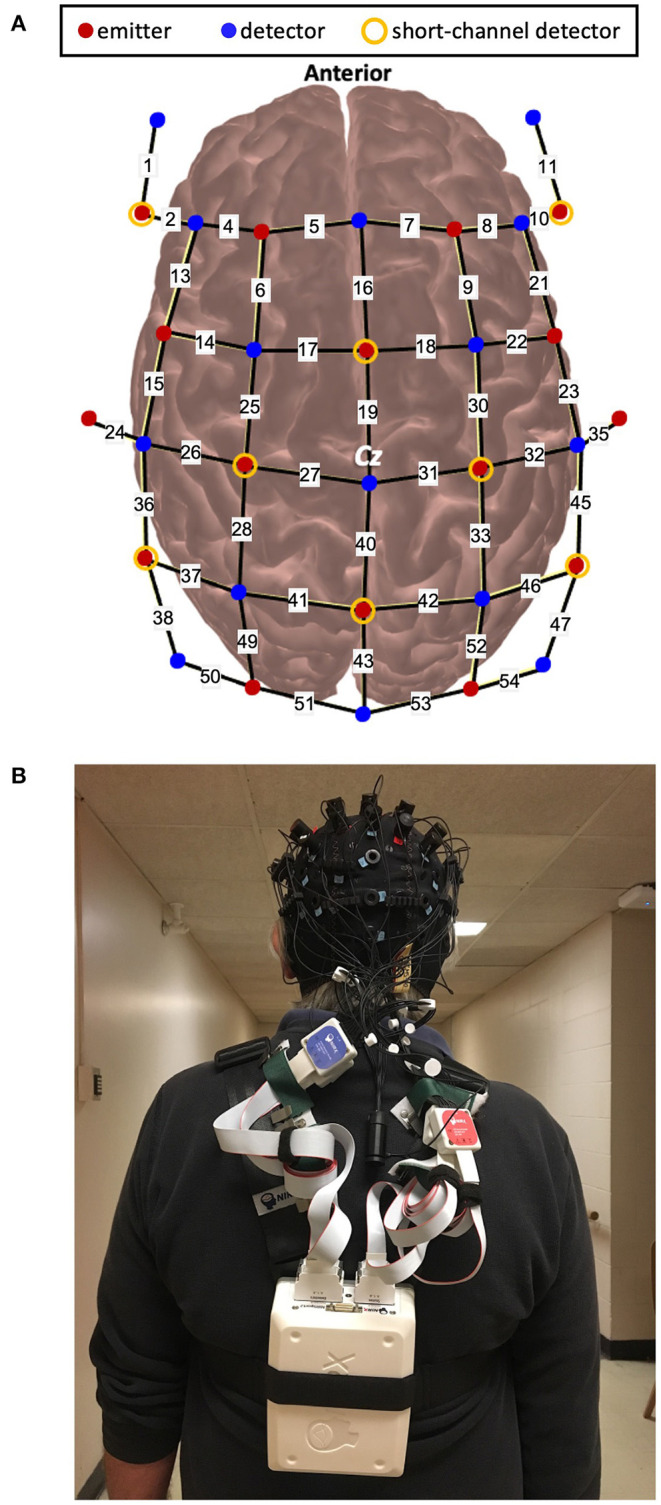
**(A)**. Schematic of optode configuration and **(B)**. Example of a participant wearing the fNIRS device.

A wireless and portable fNIRS device (NIRSport2, NIRx Medical Technology, Germany) was used. This device consists of 16 LED emitters and 23 silicon photodetectors. Emitters released near-infrared light at 760 and 850 nm which enabled measurement of both HbO and HbR. The probe configuration for this experiment arranged the emitter and detector pairs to result in 48 long separation channels (approximately 30–35 mm apart) and 8 short separation channels (8 mm apart) (Figure 1A). Two different distances were chosen to control for extracerebral contamination (24). The optodes are wired to the fNIRS collection device that was worn as a backpack by the participants. The headcap weighed <1 lb and the backpack device weighed 4 lbs (Figure 1B). fNIRS data were obtained using the software Aurora 1.4 (NIRx Medical Technologies, Berlin, Germany). For each data collection session, calibration of the fNIRS signal was completed to assess the signal quality. Channels were classified as “Excellent,” “Acceptable,” “Critical,” or “Lost.” Classifications were documented for comparison with pre-processing methods. Data were then continuously sampled at 4.36 Hz.

#### Probabilistic Localization

Several approaches were taken to improve the accuracy of localizing functional brain activation. The spatial location of the optodes on each participant's head was digitally recorded using a 3D digitizer (Polhemus Patriot, USA) and the software PHOEBE (25). To further control for the location of digitization within the optode holder (7 mm diameter), a custom interface between the optode holder and the digitizing stylus was 3D printed to consistently place the stylus at the center of the optode holder.

#### Stroke Impairment

The lower extremity portion of the Fugl-Meyer assessment (FMLE) (26) was used to evaluate motor impairment after stroke and was completed by a trained physiotherapist. This assessment has excellent inter- (27) and intra-rater (28) reliability, and is a recommended outcome measure for individuals living after stroke (29).

#### Task Performance

Performance was measured using two outcomes: gait speed and stride-time variability. Gait speed was calculated for every trial by determining the distance walked during the 30 sec trials and then calculating speed in meters per second. Stride-time variability was measured using foot pressure switches (Noraxon, USA) on the paretic leg. Specifically, four separate pressure sensors were secured to the sole of the shoe: at the heel, first metatarsal head, fifth metatarsal head, and first distal phalanx. The transmitter was secured onto the lower leg of the participant and the signal was sent to a receiver attached to the data collection laptop. Data were collected at 100 Hz.

### Task Procedure

Participants completed the walking trials along a 50-meter hallway. They were first provided with 1–2 familiarization trials of the walking condition to become accustomed to walking with the fNIRS backpack. Each trial started with the participant standing at one end of the hallway. The starting end was randomly determined by the researcher. After a minimum 30s quiet stance, a verbal “go” from the researcher indicated the start of the walking trial and a verbal “stop” indicated the end of the trial. All participants were given instructions to keep their head position consistent, avoid talking throughout the walking trials, and to walk at their normal, comfortable pace. Each walking trial was 30 s long and was performed 4–5 times. For all trials, a manual wheelchair and a spotter were positioned behind the participant for safety. At the end of each walking trial, participants stood for 5 s. To minimize fatigue associated with time spent on their feet and extra steps needed to reach the starting point of the next trial, participants were asked to sit in the wheelchair after each trial, and then pushed to the end of the hallway to start the next trial. All trials had at least 30 s of quiet standing immediately prior to the start of the trial—this allowed for the fNIRS signals to return to baseline, and a portion of this period was used for baseline comparisons. The PychoPy3.0 program was used for triggering and timing the trials (30).

### Data Processing

#### Functional Brain Activation

Preprocessing of fNIRS data was completed using an open source software, HomER2 (31). All HomER2 functions and corresponding parameters are indicated within square brackets. Noisy channels were first removed [enPruneChannels: SNRtresh = 6.67, dRange = 5e−4 to 1e+00, SDrange:0–45] and compared with the calibration from the Aurora software. Data were then converted into optical density [hmrIntensity2OD]. Motion artifacts were then identified by using 0.5s time windows to determine if the signal exceeded either 20 standard deviations above the mean signal for each channel or showed a change >100 times in amplitude [hmrMotionArtifactByChannel: tMotion = 0.5, tMask = 1.0, STDEVthresh = 20.0, AMPthresh = 5.00]. The number of removed channels for each participant can be found in Supplementary Material (Supplementary Table S2). Motion correction was then applied using a wavelet transformation with a 1.5 interquartile range [hmrMotionCorrectWavelet: iqr = 1.5] (18, 32–34). A lowpass filter of 0.15 Hz was then applied to the data [hmrBandpassFilt: lpf = 0.15] and converted to hemoglobin concentration using the modified Beer-Lambert equation [hmrOD2Conc: ppf = 6.0] (35, 36). The hemodynamic response was estimated using a general linear model with an ordinary least squares approach (37, 38) and a 0.5 s width and 0.5 s step consecutive gaussian basis function (39). Superficial contributions to the signal were also removed by regressing out the data from the short separation channel that has the highest correlation to each channel (19, 37, 39–41). Any drift within the signal was corrected using a 3rd order polynomial correction (37) [hmrDeconvHRF_DriftSS: trange = −20.0 35.0, glmSolveMethod = 1, idxBasis = 1, paramsBasis= 0.5 0.5, rhoSD_ssThresh = 15.0, flagSSmethod = 1, driftOrder = 3, flagMotionCorrect = 0].

Preprocessed data were then exported to a custom Matlab script for baseline corrections (−15 to 0 s before walking onset), averaging, and visualization of data. Each channel was visually inspected for remaining motion artifacts or non-physiological noise. The group responses were averaged across three Segments: baseline standing (Stand: −10 to 0 s), Acceleration (Accel: 0–10 s), and Steady-State (Steady: 10–30 s).

#### Probabilistic Localization

The spatial location of each participant's optode position was imported to AtlasViewer (42) and used to project the channels to the Colin27 atlas brain. Montreal Neurological Institute (MNI) coordinates and Automated Anatomical Labeling projections were provided by AtlasViewer and were then translated into Brodmann labels using the Allen Human Brain Atlas (43) and the Yale BioImage Suite Package web application (44). Using this labeling system, channels were then categorized into regions of interest: PFC, PMC (which was also combined with SMA), SMC, and PPC on the ipsilesional and contralesional hemispheres. Individual channel classifications can be found in Supplementary Material (Supplementary Table S3). Additionally, individual MRIs were used to determine location of the stroke lesion. If stroke lesions were present along the cortex, channels that were projected to lesion sites were removed.

#### Stride-Time Variability

Data were processed using a custom Matlab script (Matlab 2017b). Each stride was visually inspected and marked to determine stride-time. The first and last 2 strides of each trial were removed to account for transient stride-time changes. Stride-time variability was identified as the standard deviation around the mean for each trial, which was then averaged across all trials for each participant.

### Statistical Analyses

#### Aim 1

The group average of the hemodynamic responses was used to describe the temporal characteristics of the response. Specifically, for each region of interest, a baseline threshold was determined by calculating the baseline mean (−10 to 0 s before walking onset) then two standard deviations above and below this mean. Time points were identified when the signal deviated away from and returned to the baseline threshold and at peak positive/negative responses.

#### Aim 2

Four separate linear mixed-effects models were used to predict regional brain activation with walking for each hemoglobin species (i.e., HbO and HbR). The model included Participants as random effects and the following fixed effects were then independently tested for significant contributions to the model: Segment (Stand, Accel, Steady), Hemisphere (contralesional, ipsilesional), and Lesion Side (left, right). If an independent fixed effect significantly contributed to the model for any region of interest comparison, it was included in the final model. Interaction effects between fixed variables were included if it significantly added to the model, as assessed by the likelihood ratio test. The R package “lme4” was used for the linear mixed-effects models.

As large inter-subject variability has been previously reported in functional brain activation after stroke (45), further exploration of this variability was identified using k-means clustering with two clusters to determine the proportion of participants with distinctly different activation patterns. K-means clustering was performed for HbO and HbR separately. Data from both the Accel and Steady segments, both Hemispheres, and the four region of interests were used to determine a participant's cluster designation. K-means clustering (with twenty random starts) was computed and the clustering that occurred most frequently was used to cluster the data.

#### Aim 3

Pearson's correlations were assessed for each of the regions of interest to explore the relationship between brain activation and impairment or performance. For this aim, Accel and Steady segments were averaged together for each region of interest and correlated separately to FMLE, gait speed, and stride-time variability. Relationships with contralesional and ipsilesional activations were assessed separately, resulting in eight regions of interest. For further exploration, demographic and clinical outcome scores were also reported by clusters.

#### All Statistics Were Completed Using R Studio

All relevant assumptions and diagnostics were checked for each statistical test and appropriate modifications were made and reported if violations occurred. Given the exploratory objectives, results were reported using a standard alpha of 0.05 in order not to miss potential effects. Data are also shown with a Bonferroni correction for reducing Type 1 error by running multiple models (*p* ≤ 0.0125 = 0.05/4 models and regions) and a Benjamini-Hochberg false discovery rate of 5% to correct for multiple Pearson's correlations.

## Results

Twenty-two participants were eligible for the study and 20 participants completed the study. Two eligible participants were not able to attend data collection sessions due to restrictions as a result of the COVID-19 pandemic. Table 1 shows a summary of participant demographics. Individual participant data can be found in Supplementary Materials.

**Table 1:** Summary of participant demographics and performance.

***N* = 20**	
Age [mean (SD)]	64 (7.6) years
Sex (Female/Male)	7/13
Chronicity (months)	82 (67.4) months
Lesion Depth (cortical/subcortical/mixed)	0/17/3
Lesion side (Left/Right)	7/13
FMLE (34 max)	27 (4.9)
MoCA (30 max)	26 (2.6)
Gait Aids (none/walking stick(s)/4 point cane/4 wheeled walker)	12/6/1/1
Gait Speed [mean (SD)]	0.84 (0.345) m/s Range: 0.15–1.39 m/s
Stride-time variability of paretic leg [mean (SD)]	0.08 (0.03) sec

### Aim 1: Temporal Hemodynamic Response Profile

**Figure 2:**
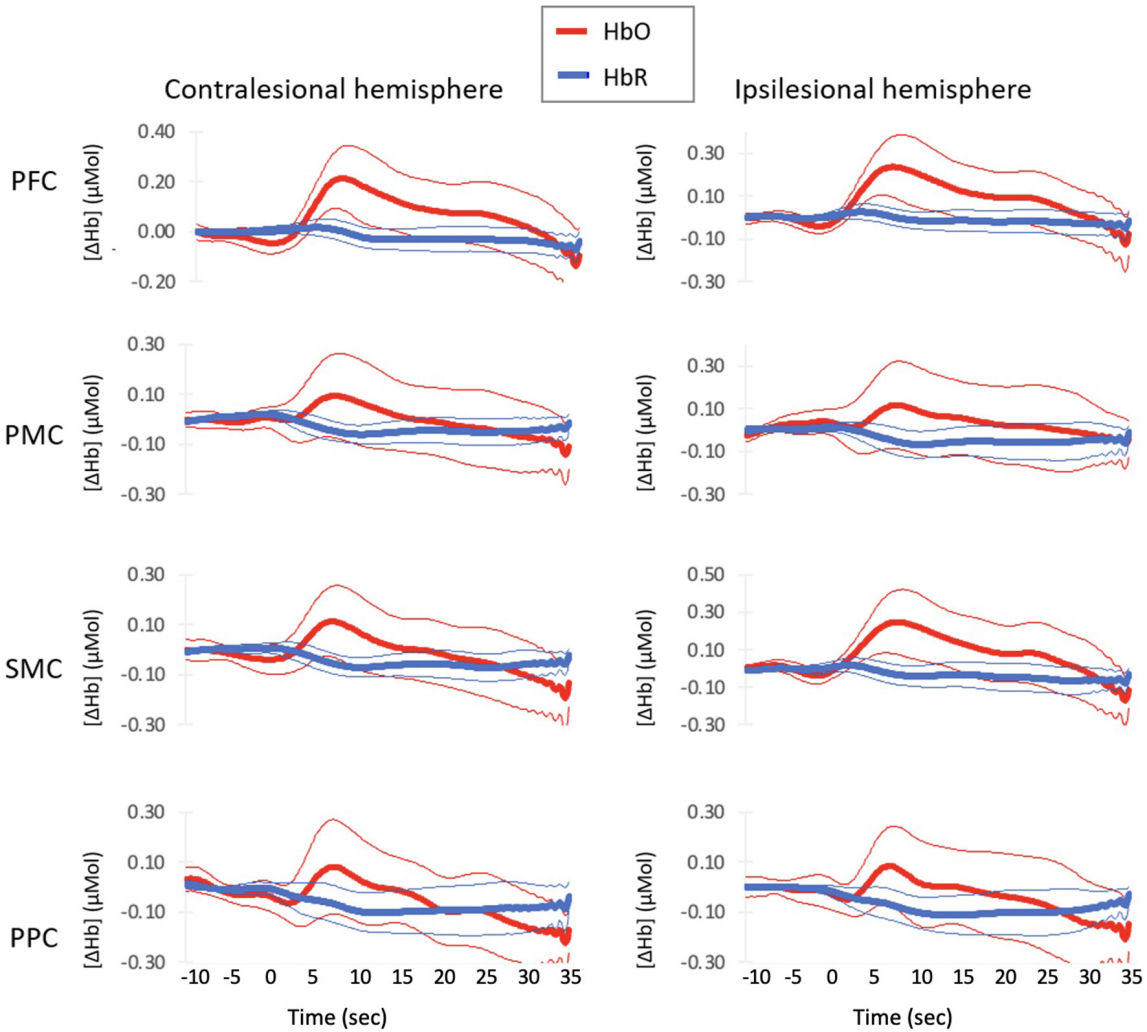
Average Hemodynamic Responses with Normal–paced Walking for each region of interest. Thick lines indicate group mean response and thin lines indicates 2 standard errors around the mean.

Group averaged hemodynamic responses are shown in Figure 2. The average hemodynamic response profile was similar for HbO across all eight regions of interest. HbO signals increased above baseline around 3.4 (1.43) sec and peaked around 7.4 (0.30) sec. For PFC, the increase in HbO remained elevated until 30.2 and 28.9 sec for the contralesional and ipsilesional side, respectively. HbO levels did not remain elevated for as long in bilateral PMC, contralesional SMC, or bilateral PPC−14.9 (3.74) sec. Ipsilesional SMC activation remained elevated for 27 seconds.

Deoxyhemoglobin profiles for bilateral PFC and ipsilesional SMC showed an initial increase in HbR starting at 0 (0.58) sec that peaked at 3.3 (0.95) sec. These regions then decreased HbR concentration starting at 7.3 (1.7) sec. Bilateral PMC, contralesional SMC, and bilateral PPC decreased starting at 1.9 (1.64) sec. All regions continued to decrease HbR concentrations until the walking task finished.

### Aim 2: Brain Activation During Walking

The best fit linear mixed-model included Participant as random effects, and Hemisphere and Segment as fixed effects. No significant effects on the model were found for Lesion side. The addition of interaction effects did not significantly add to the model. Overall, the model's total explanatory power was moderate (HbO conditional R^2^ = 0.19–0.28; HbR conditional R^2^ = 0.11–0.19) and the part related to the fixed effects alone (marginal R^2^) ranged from 0.003–0.05 for HbO and 0.02–0.03 for HbR.

**Figure 3:**
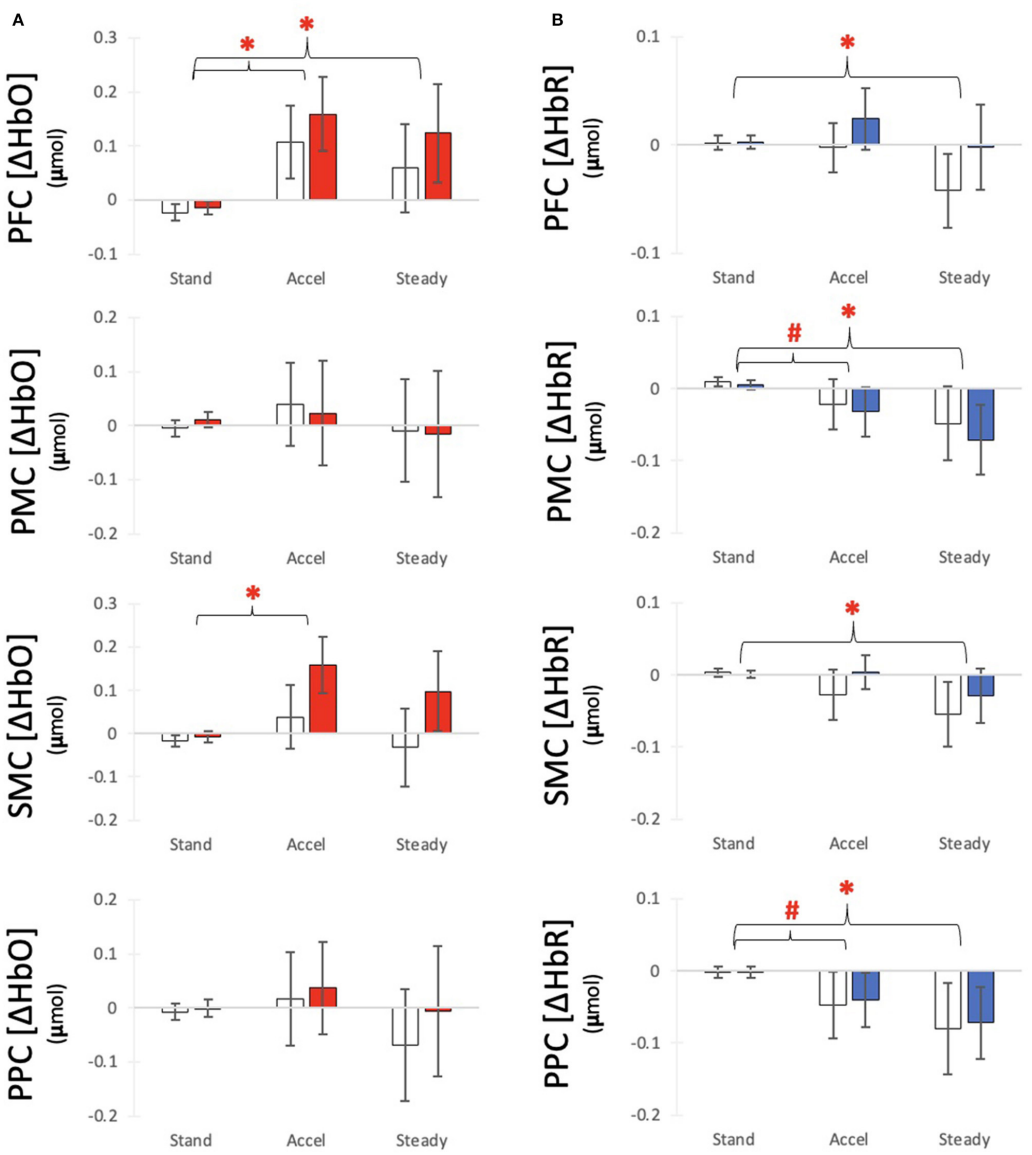
Average **(A)** HbO and **(B)** HbR response for each time segment with for each region of interest. Bars indicate average responses for the contralesional side (white) and ipsilesional side (colored) and error lines indicate standard error. ^#^Indicates a differences from Baseline with alpha at 0.05 *Indicates a significant difference from Baseline after correction for multiple comparisons.

The HbO models showed significant increases in concentration from Stand to Accel (x_diff_ = 0.153 μmol) and Steady (x_diff_ = 0.112 μmol) in PFC and during Accel only in SMC (x_diff_ = 0.104 μmol) (Figure 3A). Significant differences between hemispheres were observed for SMC with the ipsilesional hemisphere x_diff_ = 0.066 μmol (Table 2). The HbR models showed significant decreases in concentration during Steady for all regions: PFC x_diff_ = −0.023 μmol; PMC x_diff_ = 0.066 μmol; SMC x_diff_ = −0.046 μmol; PPC x_diff_ = −0.0740 μmol compared to Stand (Figure 3B). Significant differences between hemispheres were only observed for PFC with the ipsilesional hemisphere showing 0.019 μmol greater HbO than the contralesional hemisphere (Table 3). K-means clustering for HbO grouped nine participants as generally having lower functional brain activity with walking and 11 showing generally higher activation. HbR clusters resulted in 10 participants having distinctly greater HbR concentration decreases compared to the other 10 participants.

**Table 2:** Linear mixed-effects models results for HbO: HbO~Condition+Hemisphere+(1|Participant).

**ROI**	**Predictors**	**Estimates**	**Confidence interval**	***p***	**ICC**	**N_**subj**_**	**Observations**	**Marginal R^**2**^ / Conditional R^**2**^**
PFC	(Intercept)	−0.036837	−0.104026–0.030353	0.282	0.21	20	843	0.053 / 0.251
	Segment [Prep]	0.153421	0.111363–0.195479	<0.001^*^				
	Segment [Stdy]	0.111775	0.069717–0.153833	<0.001^*^				
	Hemisphere [ipsi]	0.036446	0.001732–0.071160	**0.040**				
PMC	(Intercept)	0.005330	−0.086767–0.097426	0.910	0.27	20	705	0.003 / 0.272
	Segment [Prep]	0.029432	−0.025459–0.084323	0.293				
	Segment [Stdy]	−0.014336	−0.069227–0.040555	0.608				
	Hemisphere [ipsi]	0.006386	−0.039135–0.051907	0.783				
SMC	(Intercept)	−0.037187	−0.117869–0.043496	0.366	0.26	20	465	0.032 / 0.283
	Segment [Prep]	0.103951	0.046891–0.161011	<0.001^*^				
	Segment [Stdy]	0.037538	−0.019522–0.094598	0.197				
	Hemisphere [ipsi]	0.065918	0.017822–0.114015	0.007^*^				
PPC	(Intercept)	−0.020774	−0.113255–0.071707	0.659	0.19	19	507	0.006 / 0.190
	Segment [Prep]	0.030634	−0.040401–0.101670	0.397				
	Segment [Stdy]	−0.033446	−0.104482–0.037590	0.355				
	Hemisphere [ipsi]	0.018087	−0.040443–0.076617	0.544				

*Predictors indicate the fixed effects levels within the variables in the model. Reference levels were Stand for Condition and the contralesional hemisphere for Hemisphere. Estimates indicate the adjusted difference between the reference level and the predictor level. Bolded p–values indicate significant differences using an alpha of 0.05*.

* *Indicates significant differences with p ≤ 0.0125 (0.05 /4. Bonferroni correction for four models). Estimates and confidence intervals are described in μmol. PFC, pre-frontal cortex; PMC, premotor cortex; SMC, sensorimotor cortex; PPC, posterior parietal cortex*.

**Table 3:** Linear mixed–effects models results for HbR: HbR~Condition+Hemisphere+(1|Participant).

**ROI**	**Predictors**	**Estimates**	**Confidence interval**	***p***	**ICC**	**N_**subj**_**	**Observations**	**Marginal R^**2**^ / Conditional R^**2**^**
PFC	(Intercept)	−0.005865	−0.030568–0.018839	0.641	0.15	20	843	0.020 / 0.163
	Segment [Prep]	0.009288	−0.008636–0.027212	0.309				
	Segment [Stdy]	−0.023347	−0.041271−0.005422	0.011^*^				
	Hemisphere [ipsi]	0.019295	0.004509–0.034082	0.011^*^				
PMC	(Intercept)	0.012104	−0.018544–0.042753	0.438	0.09	20	705	0.029 / 0.120
	Segment [Prep]	−0.034157	−0.061640−0.006675	**0.015**				
	Segment [Stdy]	−0.066126	−0.093608−0.038643	<0.001^*^				
	Hemisphere [ipsi]	−0.012357	−0.035100–0.010386	0.286				
SMC	(Intercept)	−0.006921	−0.035947–0.022106	0.640	0.09	20	465	0.023 / 0.113
	Segment [Prep]	−0.016582	−0.045085–0.011922	0.254				
	Segment [Stdy]	−0.045554	−0.074057−0.017051	0.002^*^				
	Hemisphere [ipsi]	0.016181	−0.007689–0.040051	0.183				
PPC	(Intercept)	−0.009275	−0.055350–0.036800	0.693	0.17	19	507	0.026 / 0.190
	Segment [Prep]	−0.041710	−0.078337−0.005084	**0.026**				
	Segment [Stdy]	−0.074196	−0.110822−0.037569	<0.001^*^				
	Hemisphere [ipsi]	−0.004291	−0.034464–0.025882	0.780				

*Predictors indicate the fixed effects levels within the variables in the model. Reference levels were Stand for Condition and the contralesional hemisphere for Hemisphere. Estimates indicate the adjusted difference between the reference level and the predictor level. Bolded p–values indicate significant differences using an alpha of 0.05*.

* *Indicates significant differences with p ≤ 0.0125 (0.05 /4: Bonferroni correction for four models). Estimates and confidence intervals are described in μmol. PFC, pre-frontal cortex; PMC, premotor cortex; SMC, sensorimotor cortex; PPC, posterior parietal cortex*.

### Aim 3: Relationship Between Brain Activation and Impairment or Performance

**Figure 4:**
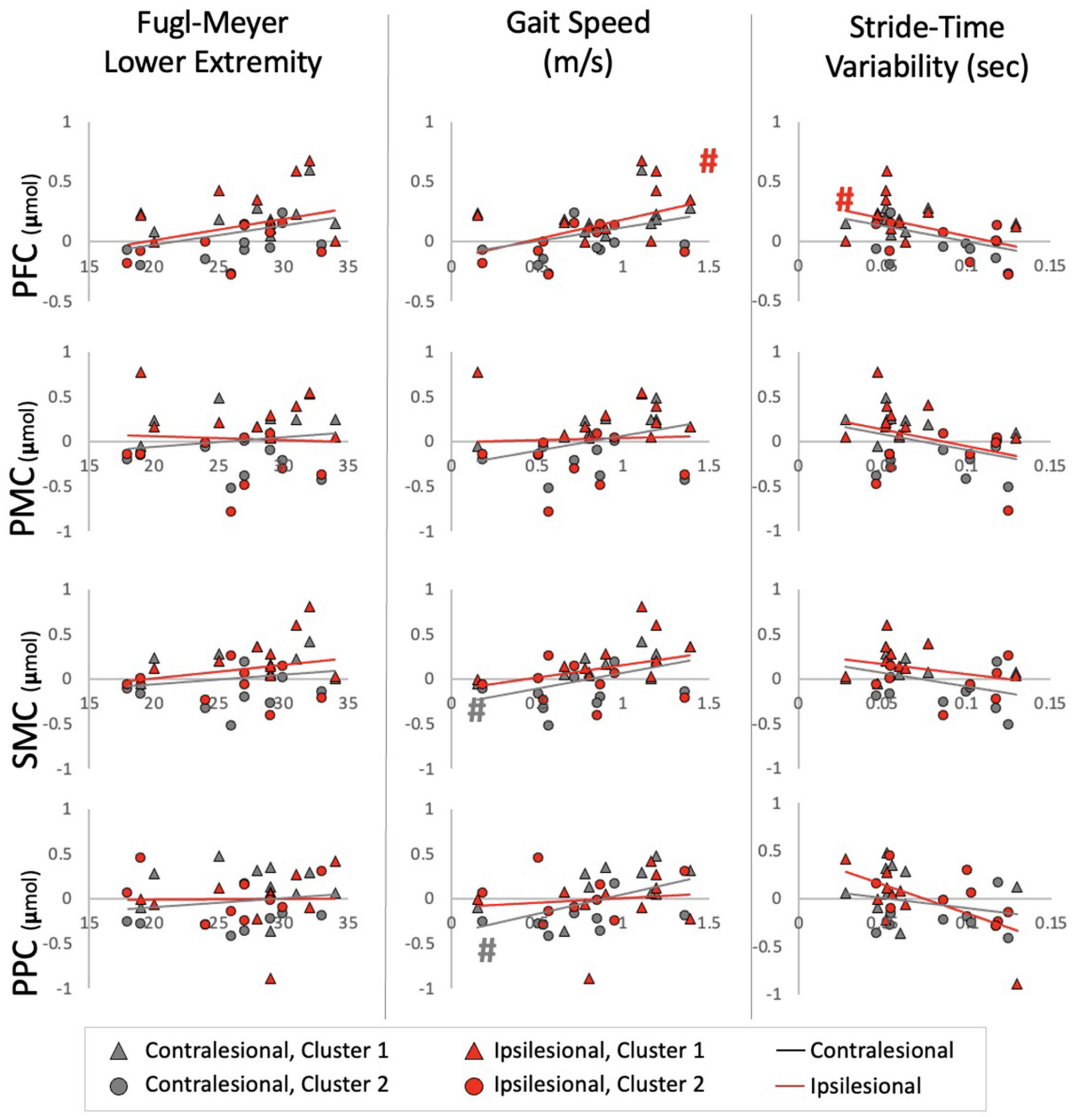
Relationships between HbO and impairment, performance, and automaticity for each ROI Ipsilesional and Contralesional hemisphere are plotted on a single graph and indicated by red and gray markers; respectively. Additionally; individuals in Cluster 1 are denoted by a triangle marker and individuals in Cluster 2 are denoted by circle markers. ^#^Indicates a relationship with alpha at 0.05.

For HbO, positive relationships were observed between Gait Speed and ipsilesional PFC (r^2^ = 0.21, p = 0.040), contralesional SMC (r^2^ = 0.26, *p* = 0.023) and contralesional PPC (r^2^ = 0.29, *p* = 0.017). A negative relationship was also observed between Stride-Time Variability and ipsilesional PFC (r^2^ = 0.29, *p* = 0.018). These correlations, however, were not significant after multiple comparison correction (Figure 4). No significant relationships were observed with lower extremity impairment (FMLE) or with any outcomes and HbR.

Further exploration of the brain activation clustering showed some observable trends in various demographic and clinical outcomes. In general, individuals in the higher HbO concentration cluster had better physical outcomes (Table 4). Minimal clinically important differences (MCID) between the two clusters were found for gait speed [MCID: 0.04–0.2 m/s (46, 47), cluster difference: 0.18 m/s] and Short Physical Performance Battery [MCID: 0.55 points (47), cluster difference: 1.9 points]. For the HbR clusters, individuals who were in the lower HbR concentration cluster (i.e., greater decrease in HbR) had overall better outcomes (Table 5).

**Table 4:** Demographic and clinical outcome averages split between HbO clusters.

	**Cluster 1 (*****N*** **=** **11)**		**Cluster 2 (*****N*** **=** **9)**		**95% Confidence**	**Cohen's D**
	**lower [HbO]**		**higher [HbO]**		**interval for difference**	**effect size**
	**MEAN**	**SD**	**MEAN**	**SD**		
Age	66	6.4	63	9.0	−4.24–10.24	0.39
Time post–stroke (months)	72	62	94.3	74.89	−86.54–41.94	0.33
Years of post–secondary education	5.3	2.88	4.6	3.78	−2.43–3.83	0.21
Number of comorbidities	0.7	1.10	1.1	1.27	−1.51–0.71	0.34
Activity–specific balance confidence	72.3	20.12	64.3	23.90	−12.66–28.66	0.37
Montreal cognitive assessment	26.7	2.97	26.4	2.07	−2.16–2.76	0.12
Short physical performance battery	9.7	2.41	7.8	3.27	−0.77–4.57	0.67
Timed up and go	16.4	13.00	19.9	12.07	−15.39–8.39	0.28
Fugl–meyer lower extremity	27.8	4.83	25.9	4.91	−2.69–6.49	0.39
Normal–pace gait speed	0.91	0.346	0.73	0.333	−0.14–0.50	0.53
Stride time variability	0.07	0.025	0.09	0.036	−0.05–0.01	0.66

**Table 5:** Demographic and clinical outcome averages split between HbR clusters.

	**Cluster 1 (*****N*** **=** **10)**		**Cluster 2 (*****N*** **=** **10)**		**95% Confidence**	**Cohen's D**
	**lower [HbR]**		**higher [HbR]**		**interval for difference**	**effect size**
	**MEAN**	**SD**	**MEAN**	**SD**		
Age	62	7.2	67	7.7	−12.00–2.00	0.67
Time post–stroke (months)	71.3	76.18	100.4	57.07	−92.34–34.14	0.43
Years of post–secondary education	5.1	2.67	4.6	4.07	−2.73–3.73	0.15
Number of comorbidities	0.8	1.03	1.0	1.41	−1.36–0.96	0.16
Activity–specific balance Confidence	68.2	23.34	68.5	22.35	−21.77–21.17	0.01
Montreal cognitive assessment	27	2.6	26	2.8	−1.54–3.54	0.37
Short physical performance battery	9	2.7	9	3.3	−2.83–2.83	0.00
Timed up and go	18.0	11.82	18.5	14.14	−12.74–11.74	0.04
Fugl–meyer lower extremity	26.9	4.65	26.6	5.39	−4.43–5.03	0.06
Normal–pace gait speed	0.78	0.300	0.88	0.394	−0.43–0.23	0.29
Stride time variability	0.08	0.038	0.07	0.024	−0.02–0.04	0.31

## Discussion

This is the first walking study completed in the stroke population that described the hemodynamic response profiles across frontal to parietal regions, showed regional activation differences, and relationships with clinical measures. Our results partially supported our hypotheses and are in-line with previous work. Some of these discrepancies may be explained by our specific participant pool and the large variability in fNIRS data across participants.

### Sustained PFC Activation and Initiation Through SMC

Based on results from 11, we hypothesized a sustained activation for all regions during the entire walking task. Contrary to our hypothesis, we only observed a sustained HbO response in PFC and significant differences between *Stand* and both *Accel* and *Steady* segments for this region only. The differences between the hemodynamic response profiles in the study by 11 and the current study may be explained by the study population (i.e., subacute stroke with ataxia compared to chronic stroke with no ataxia). During the sub-acute stage of stroke (13) or when walking with an additional challenge (34), activation in other brain regions such as the SMC are increased. This increase in motor cortex activation appears to decrease with gait recovery (13). Additionally, when walking is aided by a robotic exoskeleton, thus making walking easier, PMC and SMC activations are decreased compared to unassisted walking (48). Along these lines, our results suggest that individuals in the chronic stage of stroke, and with independent ambulation abilities, still activate the PFC throughout the walking task whereas other regions, such as the SMC, are primarily involved during the initiation and early stages of walking.

Of note, a significant decrease in HbR, which also indicates increased neural activation, was observed during steady-state walking in PFC, PMC, SMC, and PPC. Previous fNIRS studies in walking have primarily reported findings in HbO concentrations (10). Although reporting of HbR is recommended (5, 49), few studies report these findings and further interpretation of these findings is limited.

### Automaticity

#### General Trend of More Activation With Lower Stride-Time Variability (More Automatic)

Interestingly, although a significant relationship between brain activation and stride-time variability (i.e., marker of automaticity) was only found for HbO in ipsilesional PFC and PMC, the overall trend and exploratory cluster findings show some indication toward greater stride-time variability (i.e., less automatic gait) relating to decreased brain activation (Figure 4). This contrasts with our hypothesis of increased automaticity and decreased brain activation. The majority of work using stride-time variability as a marker of gait automaticity stems from the healthy older adult population (50, 51). Although others have also shown increased gait variability as indicators of poorer gait performance in the stroke population (52, 53), it's unclear if it is a marker of decreased automaticity. Further work is needed to determine measures of automaticity in the stroke population. Alternatively, greater brain activation, especially in the executive function, motor planning, and preparation areas, may be required for walking in a consistent manner. This increased activation may be a positive compensatory mechanism for better gait performance post-stroke.

### Posterior Parietal Cortex

This is the first study to explore PPC activation using fNIRS during walking post-stroke. This region is involved in upright balance (54) and postural control (55, 56). Other imaging modalities have found increased activation in this area during walking post-stroke [electroencephalography: (57–59); positron emission topography: (17)]. Additionally, work from our group primarily showed differences in the posterior parietal channels with robotic-assisted walking in healthy adults (18), highlighting the importance of measuring activation in this area. Current results showed decreased HbR concentrations in PPC with walking. No significant changes were observed for HbO though large inter-subject variations were present. The involvement of this area during walking post-stroke remains uncertain and future work with greater sample sizes or more stringent inclusion criteria may reveal the role of this area.

### Hemoglobin Response Predictions and Its Variability

Compared to standing, each region of interest showed a different response during the *Accel* and *Steady* segments of walking. Our best fit model was able to capture the differences between segments in each region of interest, but the segment and hemisphere effects only explained up to 3% of the variance within the data (marginal R^2^). This low percentage suggests other factors that may explain the variance within HbO and HbR. Our exploratory analysis point to distinct clusters of activation patterns and show some differences in physical outcomes between the clusters. This suggests that future work may want to either include more stringent inclusion criteria to reduce the variability or use physical measures as possible effect modifiers in analysis.

### Limitations

Previous reviews have acknowledged a limitation in sample sizes for fNIRS studies, with the majority including <10 or 15 participants (10, 60, 61). While this study had 20 participants, sample size calculations were estimated from studies focusing on PFC activation. This calculation was likely an underestimation of the sample size needed to observe changes in other cortical areas. Tables 2, 3 provide the necessary information for sample size calculations in future studies.

Although all our participants had observable hemiparetic gait, we acknowledge that they only represent a portion of stroke survivors. On average, our participants would be categorized in the community ambulator walking category (i.e., gait speed > 0.80 m/s) and this may explain the lack of hemispheric differences we observed. Our previous work suggests asymmetrical activation was more commonly observed in studies with an average walking speed of 0.05 m/s or slower (10). Further work in slower or more severely affected walkers is needed to further explore activation asymmetries as a marker of gait performance.

## Conclusions

Recent technologies (e.g., short-separation channels and probabilistic channel localization) and analysis methods (e.g., motion and extracerebral noise corrections) have enabled more accurate interpretation of fNIRS data. PFC continues to show consistent increases in activation during walking with the ipsilesional hemisphere showing even greater activation. SMC are active during the acceleration stage of walking and posterior parietal cortices are activated during the steady-state portion of walking. Our data also suggest that faster gait speeds related to greater brain activity in the contralesional motor and sensory regions. Our results shed light on the variability within the data, even when extraneous noise is controlled for. Inter-subject variability within functional brain data has been well reported in other brain imaging methodologies and exploratory cluster analysis in the current study revealed distinct activation responses between participants. It is likely that a larger number of participants is still needed to enable further subgroup analyses with distinct brain activation profiles.

## Data Availability Statement

The original contributions presented in the study are included in the article/Supplementary Material, further inquiries can be directed to the corresponding authors.

## Ethics Statement

The studies involving human participants were reviewed and approved by Clinical Research Ethics Board, University of British Columbia. The patients/participants provided their written informed consent to participate in this study.

## Author Contributions

SBL and JJE conceived the questions. SBL, SP, and C-lY collected the data. SBL analyzed the data and prepared the first draft of the manuscript. All authors contributed to the study design and edited the manuscript.

## Funding

The authors would like to acknowledge the funding agencies that help supported this research: University of British Columbia Four-Year Fellowship (SBL), Canadian Institute of Health Research (Fellowship for SBL and SP; Foundation Grant FND143340 to JJE), the Michael Smith Foundation for Health Research (SP), the Canada Research Chairs Program (JJE, TL-A, and LB), and the Heart and Stroke Foundation Canadian Partnership for Stroke Recovery (Post-doc award to C-lY). The funders had no role in study design, data collection, analysis, or preparation of the manuscript.

## Conflict of Interest

The authors declare that the research was conducted in the absence of any commercial or financial relationships that could be construed as a potential conflict of interest.

## Publisher's Note

All claims expressed in this article are solely those of the authors and do not necessarily represent those of their affiliated organizations, or those of the publisher, the editors and the reviewers. Any product that may be evaluated in this article, or claim that may be made by its manufacturer, is not guaranteed or endorsed by the publisher.
