# Supplementary material for: Phase-dependent Brain Activation of the Frontal and Parietal Regions During Walking After Stroke - An fNIRS Study
Source: Front Neurol. 2022 Jul 19;13:904722. doi: 10.3389/fneur.2022.904722 (PMC9343616; doi:10.3389/fneur.2022.904722)
Supplement: Supplementary file 1 [file Table_1.DOCX]

## Title: Greater frontal to parietal brain activation during acceleration and steady-state walking relates to less impairment and better performance after stroke—an fNIRS study

Supplementary Tables

**Table S1****. Individual participant demographic data**

| Participant ID | Age | Sex | Stroke chronicity (months) | Lesion side and depth | FMLE (/34) | Normal-pace gait speed (m/s) | Stride-time variability |
| --- | --- | --- | --- | --- | --- | --- | --- |
| S01 | 63 | M | 21 | R subcortical | 31 | 1.19 | 0.052 |
| S02 | 58 | M | 6 | R subcortical | 27 | 0.87 | 0.028 |
| S03 | 67 | M | 46 | R subcortical | 29 | 0.8 | 0.130 |
| S04 | 61 | F | 15 | L mixed | 29 | 0.66 | 0.046 |
| S05 | 58 | F | 21 | R mixed | 19 | 0.14 | 0.060 |
| S06 | 49 | F | 55 | R subcortical | 30 | 0.72 | 0.055 |
| S07 | 50 | F | 13 | R subcortical | 33 | 1.36 | 0.100 |
| S08 | 67 | M | 115 | L subcortical | 28 | 1.39 | 0.047 |
| S09 | 62 | F | 230 | L subcortical | 26 | 0.57 | 0.125 |
| S10 | 54 | M | 12 | R subcortical | 20 | 0.78 | 0.052 |
| S11 | 73 | M | 162 | R subcortical | 32 | 1.11 | 0.064 |
| S12 | 67 | M | 67 | R subcortical | 27 | 0.95 | Missing |
| S13 | 72 | M | 144 | R mixed | 24 | 0.54 | 0.118 |
| S14 | 63 | M | 38 | R subcortical | 29 | 0.9 | 0.055 |
| S15 | 69 | F | 159 | L subcortical | 18 | 0.18 | 0.118 |
| S16 | 72 | M | 126 | R unknown | 19 | 0.51 | 0.102 |
| S17 | 69 | M | 50 | L subcortical | 29 | 0.85 | 0.054 |
| S18 | 74 | F | 128 | R subcortical | 31 | 0.71 | 0.077 |
| S19 | 71 | M | 52 | L subcortical | 24 | 1.19 | 0.052 |
| S20 | 71 | M | 179 | L unknown | 34 | 1.16 | 0.086 |

Missing=technological difficulties with collecting this data

**Table S2: Number of channels removed for each participant**

| Participant | Prefrontal  cortex (PFC) | | Premotor  cortex (PMC) | | Sensorimotor cortex (SMC) | | Posterior parietal cortex (PPC) | | Total number of channels removed |
| --- | --- | --- | --- | --- | --- | --- | --- | --- | --- |
|  | Ipsi | Contra | Ipsi | Contra | Ipsi | Contra | Ipsi | Contra |  |
| S01 | 0/0 | 0/1 | 0/0 | 0/0 | 0/0 | 0/0 | 0/0 | 0/0 | **1** |
| S02 | 0/1 | 0/0 | 0/2 | 0/2 | 0/0 | 0/0 | 0/3 | 0/0 | **8** |
| S03 | 0/0 | 0/0 | 0/0 | 0/0 | 0/0 | 0/0 | 0/0 | 0/0 | **0** |
| S04 | 0/0 | 0/0 | 0/0 | 0/0 | 1/0 | 0/0 | 0/1 | 0/1 | **3** |
| S05 | 5/1 | 0/3 | 5/0 | 0/1 | 1/0 | 0/0 | 7/0 | 0/3 | **26** |
| S06 | 0/0 | 0/0 | 0/0 | 0/0 | 0/0 | 0/0 | 0/0 | 0/0 | **0** |
| S07 | 0/0 | 0/2 | 0/2 | 0/1 | 0/1 | 0/0 | 0/0 | 0/0 | **6** |
| S08 | 0/0 | 0/0 | 0/0 | 0/0 | 0/0 | 0/0 | 0/0 | 0/0 | **0** |
| S09 | 0/0 | 0/0 | 0/1 | 0/0 | 0/1 | 0/0 | 0/2 | 0/0 | **4** |
| S10 | 0/0 | 0/0 | 0/0 | 0/0 | 0/0 | 0/0 | 0/0 | 0/0 | **0** |
| S11 | 0/0 | 0/0 | 0/4 | 0/0 | 0/1 | 0/0 | 0/2 | 0/0 | **7** |
| S12 | 0/0 | 0/0 | 0/0 | 0/0 | 0/0 | 0/0 | 0/0 | 0/0 | **0** |
| S13 | 0/0 | 0/1 | 0/1 | 0/2 | 2/0 | 0/0 | 0/0 | 0/1 | **7** |
| S14 | 0/0 | 0/0 | 0/0 | 0/0 | 0/0 | 0/0 | 0/0 | 0/0 | **0** |
| S15 | 0/0 | 0/0 | 0/0 | 0/0 | 0/0 | 0/0 | 0/0 | 0/0 | **0** |
| S16 | 0/1 | 0/0 | 0/0 | 0/0 | 0/0 | 0/0 | 0/0 | 0/0 | **1** |
| S17 | 0/0 | 0/0 | 0/0 | 0/0 | 0/0 | 0/0 | 0/0 | 0/0 | **1** |
| S18 | 0/0 | 0/0 | 0/2 | 0/1 | 0/1 | 0/3 | 0/4 | 0/5 | **17** |
| S19 | 0/0 | 0/0 | 0/0 | 0/0 | 0/0 | 0/0 | 0/0 | 0/0 | **0** |
| S20 | 0/0 | 0/0 | 0/0 | 0/0 | 0/0 | 0/0 | 0/0 | 0/0 | **0** |

First number in each cell indicates the number of channels removed due to a stroke lesion, second number in each cell indicates channels removed during calibration/enPruneChannels, third number in each cell indicates the number of channels removed during the hmrMotionArtifactByChannel and hmrMotionCorrectWavelet steps. Ipsi=ipsilesional cortex, contra=contralesional cortex. Highlighted cells indicate regions with excluded channels.

**Table S3. Channels corresponding to each region for each participant**

| Participant | Prefrontal cortex (PFC) | | Premotor cortex (PMC) | | Sensorimotor cortex (SMC) | | Posterior parietal cortex (PPC) | |  |
| --- | --- | --- | --- | --- | --- | --- | --- | --- | --- |
|  | Ipsilesional | Contralesional | Ipsilesional | Contralesional | Ipsilesional | Contralesional | Ipsilesional | Contralesional | |
| S01 | **8 channels:**  7; 8; 9; 10; 11; 16; 21; 22 | **7 channels:**  1; 2; 4; 5; 6; 13; ~~14~~ | **7 channels:** 18; 19; 23; 30; 31; 32; 40 | **7 channels:**  15; 17; 24; 25; 26; 27; 28 | **4 channels:** 33; 35; 45; 46 | **3 channels:**  41; 36; 37 | **6 channels:**  42; 43; 47; 52; 53; 54 | **4 channels:**  38; 49; 50; 51 | |
| S02 | **8 channels:**  7; 8; 9; 10; 11; 16; ~~21;~~ 22 | **8 channels:**  1; 2; 4; 5; 6; 13; 14; 17 | **6 channels:** 18; ~~23; 30~~; 31; 32; 33 | **7 channels:**  15; 19; ~~25;~~ 26; ~~27;~~ 28; 40 | **3 channels:** 35; 45; 46 | **4 channels:** 24; 41; 36; 37 | **6 channels:**  42; 43; 47; ~~52; 53; 54~~ | **4 channels:**  38; 49; 50; 51 | |
| S03 | **8 channels:**  7; 8; 9; 10; 11; 16; 21; 22 | **7 channels:**  1; 2; 4; 5; 6; 13; 17 | **6 channels:** 18; 23; 30; 31; 32; 35 | **8 channels:**  14; 15; 19; 25; 26; 27; 28; 40 | **3 channels:** 33; 45; 46 | **4 channels:** 24; 36; 37; 41 | **5 channels:**  42; 47; 52; 53; 54 | **5 channels:**  38; 43; 49; 50; 51 | |
| S04 | **6 channels:**  1; 2; 4; 14; 13; 6 | **8 channels:**  5; 7; 8; 9; 10; 11; 16; 21; 22 | **5 channels:** 15; 17; 25; 26; 27 | **6 channels:**  18; 19; 23; 30; 31; 32 | **6 channels:** ~~28;~~ 36; 40; 24; 37; 41 | **3 channels:** 33; 35; 45 | **5 channels:**  38; 43; 49; ~~50;~~ 51 | **6 channels:**  42; 46; 47; ~~52;~~ 53; 54 | |
| S05 | **7 channels:**  7; ~~8; 10; 11; 16 21; 22~~ | **5 channels:**  1; ~~2;~~ 4; 5; ~~13~~ | **7 channels:** 18; 19; ~~23; 30; 31; 32; 35~~ | **7 channels:**  ~~6;~~ 14; 15; 17; 25; 26; 27 | **1 channel:**  ~~33~~ | **5 channels:** 24; 28; 36; 37; 40 | **8 channels:**  43; ~~42; 45; 46; 47; 52; 53; 54~~ | **6 channels:**  ~~38;~~ 41; 43; 49; ~~50; 51~~ | |
| S06 | **8 channels:**  7; 8; 9; 10; 11; 16; 21; 22 | **5 channels:**  1; 2; 4; 5; 13 | **7 channels:** 18; 19; 23; 30; 31; 32; 35 | **10 channels:**  6; 14; 15; 17; 24; 25; 26; 27; 28; 40 | **3 channels:** 33; 42; 45 | **2 channels:** 36; 37 | **5 channels:**  46; 47; 52; 53; 54 | **6 channels:**  38; 41; 43; 49; 50; 51 | |
| S07 | **7 channels:**  7; 8; 10; 11; 16; 21; 22 | **6 channels:**  ~~1~~; 2; 4; 5; ~~6;~~ 13 | **7 channels:**  9; 18; 19; 23; ~~30; 31;~~ 32 | **7 channels:**  ~~14;~~ 15; 17; 25; 26; 27; 40 | **4 channels:** ~~33;~~ 35; 45; 46 | **5 channels:** 24; 28; 36; 37; 41 | **5 channels:**  ~~42;~~ 47; 52; 53; 54 | **5 channels:**  38; 43; 49; 50; 51 | |
| S08 | **6 channels:**  1; 2; 4; 5; 6; 13 | **7 channels:**  7; 8; 10; 11; 16; 21; 22 | **7 channels:** 14; 15; 17; 25; 26; 27; 40 | **7 channels:**  9; 18; 19; 23; 30; 31; 32 | **4 channels:** 24; 28; 36; 37 | **5 channels:** 33; 35; 42; 45; 46 | **6 channels:**  38; 41; 43; 49; 50; 51 | **4 channels:**  47; 52; 53; 54 | |
| S09 | **6 channels:**  1; 2; 4; 5; 6; 13 | **8 channels:**  7; 8; 9; 10; 11; 16; 21; 22 | **7 channels:** 14; 15; ~~17;~~ 25; 26; 27; 40 | **6 channels:**  18; 19; 23; 30; 31; 32 | **4 channels:** 24; ~~28;~~ 36; 37 | **5 channels:** 33; 35; 42; 45; 46 | **5 channels:**  38; ~~41;~~ 49; 50; ~~51~~ | **5 channels:**  43; 47; 52; 53; 54 | |
| S10 | **8 channels:**  7; 8; 9; 10; 11; 16; 21; 22 | **7 channels:**  1; 2; 4; 5; 6; 13; 17 | **6 channels:** 18; 19; 23; 30; 31; 32 | **6 channels:**  14; 15; 25; 26; 27; 28 | **4 channels:** 33; 35; 45; 46 | **5 channels:** 24; 36; 37; 40; 41 | **5 channels:**  42; 47; 52; 53; 54 | **5 channels:**  38; 43; 49; 50; 51 | |
| S11 | **8 channels:**  7; 8; 9; 10; 11; 16; 21; 22 | **7 channels:**  1; 2; 4; 5; 6; 13; 17 | **7 channels:**  18; ~~19;~~ 23; ~~30;~~ ~~31;~~ 32; ~~33~~ | **7 channels:**  14; 15; 25; 26; 27; 28; ~~40~~ | **3 channels:** 35; 45; 46 | **4 channels:** 24; 36; 37; 41 | **6 channels:**  42; ~~43;~~ 47; 52; ~~53;~~ 54 | **4 channels:**  38; 49; 50; 51 | |
| S12 | **8 channels:**  7; 8; 9; 10; 11; 16; 21; 22 | **6 channels:**  1; 2; 4; 5; 6; 13 | **7 channels:** 18; 19; 23; 30; 31; 32; 33 | **8 channels:**  14; 15; 17; 25; 26; 27; 28; 40 | **4 channels:** 35; 42; 45; 46 | **4 channels:** 24; 36; 37; 41 | **5 channels:**  43; 47; 52; 53; 54 | **4 channels:**  38; 49; 50; 51 | |
| S13 | **7 channels:**  7; 8; 9; 10; 11; 21; 22 | **8 channels:**  1; 2; 4; 5; ~~6;~~ 13; 16; 17 | **5 channels:** 18; 23; ~~30;~~ 31; 32 | **7 channels:**  14; 15; ~~19;~~ 25; 26; 27; ~~40~~ | **4 channels:** 33; 35; ~~45; 46~~ | **5 channels:** 24; 28; 36; 37; 41 | **6 channels:**  42; 43; 47; 52; 53; 54 | **4 channels:**  38; ~~49;~~ 50; 51 | |
| S14 | **8 channels:**  7; 8; 9; 10; 11; 16; 21; 22 | **8 channels:**  1; 2; 4; 5; 6; 13; 14; 17 | **6 channels:** 18; 19; 23; 30; 31; 32 | **5 channels:**  15; 25; 26; 27; 40 | **4 channels:** 33; 35; 42; 45 | **5 channels:** 24; 28; 41; 36; 37 | **5 channels:**  43; 47; 52; 53; 54 | **4 channels:**  38; 49; 50; 51 | |
| S15 | **8 channels:**  1; 2; 4; 5; 6; 13; 14; 17 | **8 channels:**  7; 8; 9; 10; 11; 16; 21; 22 | **7 channels:** 15; 24; 25; 26; 27; 28; 40 | **7 channels:**  18; 19; 23; 30; 31; 32; 33 | **3 channels:** 36; 37; 41 | **5 channels:** 35; 42; 45; 46; 52 | **4 channels:**  38; 49; 50; 51 | **4 channels:**  43; 47; 53; 54 | |
| S16 | **9 channels:**  5; 7; 8; 9; 10; ~~11;~~ 16; 21; 22 | **5 channels:**  1; 2; 4; 6; 13 | **6 channels:** 18; 19; 23; 30; 31; 32 | **6 channels:**  14; 15; 17; 25; 26; 27 | **3 channels:** 33; 35; 45 | **6 channels:**  24; 28; 36; 37; 40; 41 | **6 channels:**  42; 46; 47; 52; 53; 54 | **5 channels:**  38; 43; 49; 50; 51 | |
| S17 | **9 channels:**  1; 2; 4; 5; 6; 13; 14; 16; 17 | **7 channels:**  7; 8; 9; 10; 11; 21; 22 | **5 channels:** 15; 25; 26; 27; 28; 40 | **7 channels:**  18; 19; 23; 30; 31; 32; 33 | **4 channels:** 24; 37; 36; 41 | **5 channels:**  35; 42; 45; 46; 52 | **4 channels:**  38; 49; 50; 51 | **4 channels:**  43; 47; 53; 54 | |
| S18 | **10 channels:**  5; 7; 8; 9; 10; 11; 16; 18; 21; 22 | **5 channels:**  1; 2; 4; 6; 13 | **5 channels:** 23; 30; ~~31;~~ 32; ~~33~~ | **9 channels:**  14; 15; 17; 19; 25; 26; ~~27~~; 28; 40 | **4 channels:** 35; 45; ~~42;~~ 46 | **4 channels:**  24; ~~36; 37; 41~~ | **4 channels:**  ~~47;~~ ~~52; 53; 54~~ | **5 channels:**  ~~38; 43; 49; 50; 51~~ | |
| S19 | **7 channels:**  1; 2; 4; 6; 13; 14; 17 | **9 channels:**  5; 7; 8; 9; 10; 11; 16; 21; 22 | **5 channels:** 15; 25; 26; 27; 28 | **6 channels:**  18; 19; 23; 30; 31; 32 | **4 channels:** 24; 36; 37; 41 | **6 channels:**  33; 35; 40; 42; 45; 46 | **4 channels:**  38; 49; 50; 51 | **5 channels:**  43; 47; 52; 53; 54 | |
| S20 | **8 channels:**  1; 2; 4; 5; 6; 13; 14; 17 | **8 channels:**  7; 8; 9; 10; 11; 16; 21; 22 | **5 channels:** 15; 25; 27; 28; 40 | **5 channels:**  18; 19; 23; 30; 31 | **4 channels:** 24; 26; 36; 37 | **5 channels:**  32; 33; 35; 45; 46 | **6 channels:**  38; 41; 43; 49; 50; 51 | **5 channels:**  42; 47; 52; 53; 54 | |

Highlighted cells indicate regions with excluded channels.
